# A field tool for prediction of body fat in Sri Lankan women: skinfold thickness equation

**DOI:** 10.1186/s41043-016-0069-6

**Published:** 2016-09-30

**Authors:** Indu Waidyatilaka, Angela de Silva, Maduka de Lanerolle-Dias, Sunethra Atukorala, Pulani Lanerolle

**Affiliations:** 1 Department of Biochemistry and Molecular Biology, Faculty of Medicine, University of Colombo, Kynsey Road, Colombo 8, Sri Lanka; 2 Department of Physiology, Faculty of Medicine, University of Colombo, Kynsey Road, Colombo 8, Sri Lanka

**Keywords:** SFT, Skinfold thickness, Fat mass, FM, Body fat, Asian, Body composition

## Abstract

**Background:**

Valid skinfold thickness (SFT) equations for the prediction of body fat are currently unavailable for South Asian women and would be a potentially robust field tool. Our aim was to assess the validity of existing SFT equations against deuterium (^2^H_2_O) dilution and, if invalid, to develop and validate an SFT equation for % fat mass (%FM) in Sri Lankan women.

**Methods:**

H_2_O dilution was used with Fourier transform infrared (FTIR) spectroscopy as the criterion method for the assessment of %FM in urban Sri Lankan women (30–45 years). This data was used to assess the validity of available SFT equations and to generate and validate a new SFT equation for the prediction of %FM against the criterion method. Women (*n* = 164) were divided into validation and cross-validation groups for the development and validation of the new equation. The level of agreement between the %FM calculated by the final derived prediction equation and the %FM obtained by ^2^H_2_O dilution was assessed using Pearson’s correlation coefficient (R) and Bland Altman plots. Student’s *t* test was used to assess over- or underestimation, and significance was set at *p* < 0.05.

**Results:**

Existing equations significantly (*p* < 0.001) underestimated %FM compared with the ^2^H_2_O dilution method. The final equation obtained was %FM = 19.621 + (0.237*weight) + (0.259*triceps). When compared with ^2^H_2_O dilution, %FM by the equation was not significantly different. There was a significant (*p* < 0.001) correlation between %FM by the reference method and %FM by the equation. The limit of agreement by Bland Altman plot was narrow with a small mean positive bias.

**Conclusions:**

Existing SFT equations were not applicable to this population. The new equation derived was valid. We report a new SFT equation to predict %FM in women of South Asian ancestry suitable for field use.

## Background

Obesity is a major contributor to the global burden of disease with the prevalence of overweight and obesity increasing manyfold in recent years in Asia [1]. The projection for the next decade is that the largest proportional increase in overweight and obesity prevalence would be in low income and middle income countries including Asia [2]. Body mass index (BMI) has been the primary method used to determine body adiposity and is currently used to categorize overweight and obesity among adults [3]. Despite its limitations in both the assessment of body fat as opposed to lean body mass as well as in screening for risk of cardiovascular disease, BMI continues to be in widespread use. The reasons for this are twofold; the ease of use in field settings and the lack of validated, accurate field methods to assess body fat. The evidence for body fat related morbidity and mortality and the rise in non-communicable disease prevalence in Asia is strong, emphasizing the requirement for such body fat assessment methods to be developed and used over BMI [4]. Asian populations are at risk of disease at lower BMI values than global cutoff values [5] and Asians have three to 5 % higher total body fat when compared with Europeans of the same BMI [6].

Subcutaneous fat is a valid indicator of both regional and total fat mass (FM) and also insulin resistance [7] with associations with cardiometabolic risk being demonstrated [8]. Skinfold thickness (SFT) is a valid method in the measurement of body density, FM, fat free mass (FFM), and their changes with time, when carried out by a trained observer using a standard protocol [9]. Skinfold thickness, due to its simplicity and low cost requiring only a skinfold caliper and training, makes it suitable for field use. The most widely used prediction equations for the determination of body fat in women are Siri equation [10] and Brozek et al. equation. [11]. Since Siri and Brozek et al. equations require a prior body density prediction, Durnin and Wormersley [12] density equation was used. Deuterium dilution method is considered the gold standard method of estimation of total body water (TBW). Fourier transform infrared (FTIR) spectroscopy has previously been shown to be a robust estimation method of detection of deuterium in saliva. Hence, deuterium dilution determined by FTIR can be used in validation of SFT equations for prediction of %FM [13].

There is much evidence that existing SFT prediction equations are population specific [13, 14]. Presently, there are no published SFT equations for the prediction of %FM in South Asian adults, with existing prediction equations being developed in Western populations. Our aim was to validate existing equations against the gold standard method of deuterium dilution and, if found invalid, to develop and validate a field tool for body fat assessment using skinfold measurements and anthropometry in Sri Lankan women.

## Methods

### Study participants

Body fat data of 164 women aged 30–45 years were used for the validation of existing equations and for the development and validation of a new SFT equation for the assessment of %FM. The women were part of a larger study on body composition and glycaemic status which recruited 617, urban Sri Lankan women without a previous history of non-communicable diseases, from the community.

The study design of this larger study, published elsewhere, used a random cluster sampling method using the smallest administrative unit within the Colombo Municipal Council area as a cluster [15].

### Anthropometry

All anthropometric measurements were measured by one trained researcher using the same equipment in order to minimize variability, under standard conditions according to the International Society for Advancement of Kineanthropometry (ISAK) protocol [16]. All measurements were taken from the left side of the body. Height and weight were recorded while the subjects were standing barefoot. Weight was measured to the nearest 0.1 kg using a calibrated electronic scale (Seca 813). Height was measured to the nearest 0.1 cm using a Stadiometer (Seca 225, telescopic height measurement), after placement of heel, buttocks, back of shoulder and occiput in the vertical plane and head in horizontal Frankfurt plane. Waist circumference was measured in standing position at the end of normal expiration, in the horizontal plane at the level of the narrowest point between the lower costal border and the iliac crest, to the nearest 0.1 cm using a non-stretchable measuring tape (Seca 200). The World Health Organization (WHO) recommended waist circumference cutoff for high risk of metabolic complications (>80 cm) was used to categorize women [5]. All measurements were taken in duplicate and the mean was calculated. Skinfold thickness (SFT) was measured using a Harpenden Caliper (graduation 0.2 mm, range 80 mm, Model: HSK-BI). Biceps, triceps, subscapular, and suprailiac (also known as iliocristale) SFTs were measured to the closest 1 mm. The skin at the appropriate site was pinched to raise a double layer of skin and the underlying adipose tissue but not the muscle. The calipers were then applied 1 cm below and at right angles to the pinch and a reading in millimeters (mm) taken 2 s later. The mean of two measurements was calculated. If the two measurements differed by 0.5 mm, a third measurement was taken and the median value was used [16].

### ^2^H_2_O dilution technique for body composition analysis

^2^H_2_O doses (Cambridge Isotope Laboratories Inc., MA, USA) were previously prepared (30 g each weighed to the nearest 0.01 g) and stored in wide mouthed leak-proof screw capped bottles at 4 °C. Bottles were not reused. Following an overnight fast and on an empty bladder, all measurements were carried out between 8.00 am and 12.00 noon at room temperature, with the participants at rest. The baseline saliva sample was collected to assess basal ^2^H_2_O concentration in the body prior to oral administration of the ^2^H_2_O dose. Administration was supervised and ingestion of the entire dose was ensured. A second and a third saliva sample was obtained at 3 and 3.5 h post administration, allowing equilibration with body water and mean enrichment was used for further calculations [17]. Entire procedure and quality control were done according to the International Atomic Energy Agency protocol [18].

Saliva samples were collected, transported at 4 °C and stored at –20 °C pending analysis. All saliva samples were centrifuged at room temperature prior to measuring enrichment, with each sample being measured in duplicate. Quality control was in accordance with International Atomic Energy Agency (IAEA) guidelines. The equation proposed by IAEA for use as a quality control measure is TBW (kg) = 7.4 × height^3^ (m^3^), where TBW from each post-dose sample is used with the height of each participant. Since this equation was not derived specifically for our population, the range incorporating the 95 % confidence interval for the above relationship (<5.7 × height ^3^or >9.6 ×height ^3^) which has also been proposed by the IAEA for assessment of accuracy was used. Using this criterion, all samples fell within the range and were therefore included. Isotope analysis was carried out in duplicate for each sample using FTIR (8400S; Shimadzu, Vienna, Austria) at St John’s Research Institute, Bangalore, India.

### Statistical analysis

Data of 164 women were included in the analysis. Statistical analysis was carried out using Statistical Package for Social Sciences (SPSS) version 20.

#### Applicability of selected SFT equations to predict %FM of healthy adult women

From published equations for females, three equations were selected, with one being for South Asian females. These selected equations were tested by comparing %FM obtained through each equation with %FM obtained by the gold standard ^2^H_2_O dilution method. Initially density was calculated using Durnin and Womersley equation from the skinfold values of the 164 women (Table 1). These density values were applied independently to Siri and Brozek et al equations to derive %FM. Percentage FM was also calculated by the equation developed by de Lanerolle-Dias et al using the skinfold thickness data from the women (Table 1). Pearson’s rank correlation coefficients were used to assess the association between %FM obtained from ^2^H_2_O dilution and %FM obtained from the above equations. Mean values for %FM by ^2^H_2_O dilution were compared with %FM values by the equations using Student *t* test for over or underestimation. The FM% cut off of >35 % for Asian women was used [19].

**Table 1:** Published SFT prediction equations

Equation	Author	Population to which the equation was derived
*D* = 1.142 − 0.0544 (log sum of bi + tric + subs + supra)	Durnin and Womersley (1974) [12]	Age 16–72 years (male/female). Stratified according to age (30–39 years) and gender. Glasgow
%FM = 495/D − 450	Siri (1961) [10]	Adults
%fat = (457/body density) − 414.2	Brozek et al (1963) [11]	Adults
%FM = 9.701 − (0.460)*age + (0.640) * SFT-triceps + (0.583) * SFT-Supra iliac	de Lanerolle-Dias et al (2011) [13]	Age 15–19 years. Adolescent girls. Sri Lanka

#### Derivation of a population-specific SFT equation to predict %FM using ^2^H_2_O dilution as the reference method

Data from the 164 women were randomly divided into validation (60 %; *n* = 100) and cross-validation (40 %; *n* = 64) groups by the statistical package (SPSS) for derivation and validation of SFT equations [13, 20]. The preliminary prediction equation for %FM was derived for the validation group, by linear regression analysis, using forward likelihood ratio, with %FM by ^2^H_2_O dilution as the dependant variable and weight, waist circumference (WC), SFT-biceps, SFT-triceps, SFT-subscapular, and SFT-suprailiac as independent variables. Predictability of the preliminary equation was evaluated using the cross-validation group. The final prediction equation was derived after combining both validation and cross-validation groups, by linear regression analysis using the enter method [13, 14, 17]. The independent variables for the final equation were determined by the preliminary equation and %FM by ^2^H_2_O dilution was used as the dependent variable. Level of agreement between the final derived prediction equation and the relevant ^2^H_2_O dilution value was assessed using Pearson’s correlation coefficient (R) and Bland Altman plots. Student’s *t* test was used to assess over or underestimation and significance was set at *p* < 0.05.

## Results

The mean age of women was 37.4 ± 3.5 years and their body composition parameters are given in Table 2.

**Table 2:** Body composition parameters of women (*n* = 164)

	Mean ± SD	Range
Height (cm)	154.4 ± 5.4	140.0–166.0
Weight (kg)	56.7 ± 9.8	32.0–84.8
Body mass index (kg/m^2^)	23.9 ± 4.1	15.2–35.9
Waist circumference (cm)	74.6 ± 8.7	52.1–98.6
Fat mass by ^2^H_2_O dilution (kg)	21.4 ± 6.0	6.7–43.9
Fat free mass by ^2^H_2_O dilution (kg)	35.3 ± 5.0	25.2–52.6
Fat mass % by ^2^H_2_O dilution (%)	37.2 ± 5.2	20.94–51.80

### Applicability of selected published SFT equations to predict %FM in urban adult women

Selected published prediction equations were tested in the 164 women (Table 1) to validate the %FM calculated by these equations against the reference method. Density obtained by Durnin and Womersley equation was applied to Siri and Brozek et al equations to derive %FM. Percentage FM derived by these two equations and the equation developed by de Lanerolle-Dias et al. significantly (*p* < 0.001) underestimated %FM by the ^2^H_2_O dilution in this population (Table 3).

**Table 3:** Percentage FM calculated using selected equations and by the Deuterium dilution method

Method	Mean ± SD	Standard error of the mean	Correlation with ^2^H_2_O dilution method value (Pearson’s correlation coefficient)
Durnin and Womersley^a^			
Siri	26.60 ± 3.7^*^	0.29	0.624^**^
Brozek et al	25.82 ± 3.4^*^	0.27	0.624^**^
de Lanerolle-Dias et al.	20.10 ± 9.2^*^	0.72	0.562^**^
%FM by ^2^H_2_O dilution	37.24 ± 5.2	0.41	

^a^Density obtained by Durnin and Womersley was applied to Siri and Brozek et al. equations

*Significant under estimation from reference method (*p* < 0.001)

**Significant correlation (*p* < 0.001)

### Derivation and validation of a SFT prediction equation for %FM in normoglycaemics

In deriving a new population-specific SFT equation for the prediction of %FM, FM data by the ^2^H_2_O dilution method was used as the reference. Skinfold thickness measurements and correlations with %FM from the criterion method are given below (Table 4).

**Table 4:** Skinfold thickness (SFT) measurements in women (*n* = 164) and correlation of SFT with %FM by the criterion method

	Mean ± SD	Range	Correlation with %FM by ^2^H_2_O method
SFT-biceps (mm)	11.03 ± 5.16	3.8–39.8	0.471^*^
SFT-triceps (mm)	16.16 ± 6.14	6.8–47.2	0.518^*^
SFT-subscapular (mm)	27.23 ± 9.8	9.1–66.8	0.580^*^
SFT-suprailiac (mm)	29.57 ± 9.39	7.8–56.6	0.559^*^

*SFT* skinfold thickness, *FM* fat mass

*Significant correlation coefficient (*p* < 0.001)

Using the sample of 164 women for the validation and cross validation, the following analysis yielded the derivation of the SFT equation. The validation (*n* = 100) and the cross-validation (*n* = 64) groups were not significantly different in age, anthropometry, and body composition data by the reference method. Percentage FM assessed by the reference method was significantly correlated with weight (*R* = 0.590, *p* < 0.001), SFT-biceps (*R* = 0.471, *p* < 0.001), SFT-triceps (*R* = 0.518, *p* < 0.001), SFT-subscapular (*R* = 0.580, *p* < 0.001), and SFT-suprailiac (*R* = 0.559, *p* < 0.001). Using %FM by the ^2^H_2_O dilution method as the dependant variable and weight, WC, SFT-biceps, SFT-triceps, SFT-subscapular, and SFT-suprailiac as the independent variables, a preliminary prediction equation was derived for %FM using the validation group. Age was not included as an independent variable as age range was narrow in this population. Of the two models obtained by linear regression analysis, the second model had a higher *R*^2^ (model 1: *R*^2^ = 0.557; model 2: *R*^2^ = 0.609) and a lower standard error of estimate (SEE) (model 1, 4.21; model 2, 4.05) and was selected. While SFT-biceps, subscapular and suprailiac were not significant predictors of %FM in this population, weight (*P* = <0.001) and SFT-triceps (*P* = 0.003) were reliable independent variables. The preliminary equation obtained was %FM = 20.037 + (0.227 * weight) + (0.257 * triceps). When the preliminary equation was applied to the cross-validation group (*n* = 64), it was significantly (*p* < 0.001) correlated (*r* = 0.693) with %FM by the ^2^H_2_O dilution method (Table 5). There was no significant difference between %FM by the ^2^H_2_O dilution method and %FM by the preliminary equation (Table 5).

**Table 5:** Preliminary and final prediction model equations

	Preliminary equation (*n* = 64)	Final equation (*n* = 164)
Equation	%FM = 20.037 + (0.227 * weight) + (0.257*triceps)	%FM = 19.621 + (0.237 * weight) + (0.259*triceps)
*R*	0.693	0.647
%FM (equation) (mean ± SD)	37.22 ± 3.6*	37.25 ± 3.4*
%FM by ^2^H_2_O dilution-reference method (mean ± SD)	37.62 ± 5.5	37.24 ± 5.2

*FM* fat mass, *SFT* skinfold thickness

^*^Significant correlation coefficient with the reference method (*p* < 0.001)

The final equation was derived following the combination of both the validation and cross-validation groups (*n* = 164). Linear regression analysis was carried out using %FM by the ^2^H_2_O dilution method as the dependant variable and weight and SFT-triceps (identified as the most predictive variables from the preliminary equation) as the independent variables. The final equation obtained was %FM = 19.621 + (0.237 * weight) + (0.259 * triceps). There was no significant difference between %FM by the ^2^H_2_O dilution method and %FM by the final equation, and %FM by the final equation was significantly (*p* < 0.001) correlated (*r* = 0.647) with %FM by the ^2^H_2_O dilution method (Table 5).

**Fig. 1:**
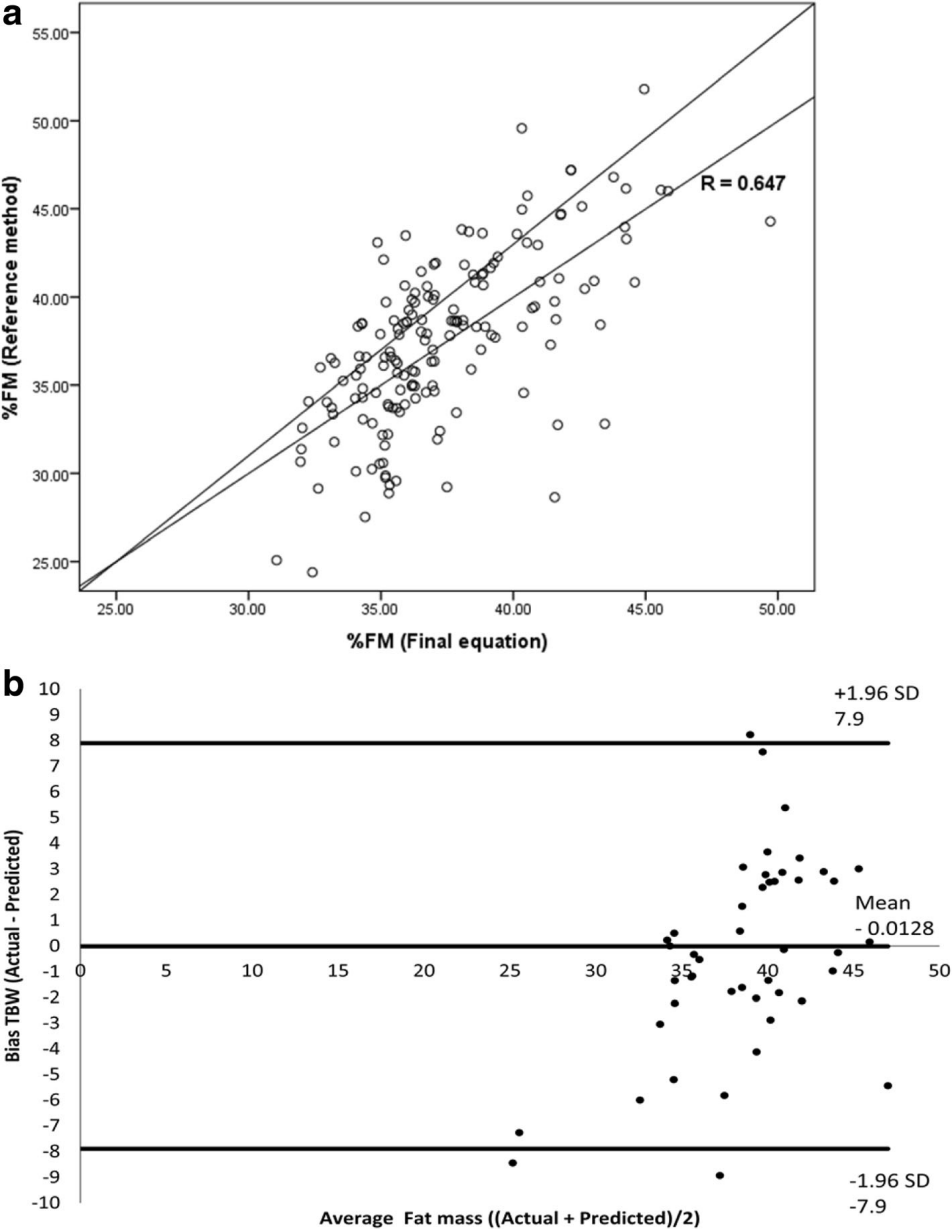
Plot of %FM by the reference method and %FM by the final equation. **a** Regression line for %FM and **b** Bland Altman plot difference for %FM

Visual comparison by scatter plots of the final equation with the reference method indicates high correlation (Fig. 1a). The Bland Altman plot between the final prediction equation and %FM by the reference method for plot difference is shown in Fig. 1b. The limits of agreement was narrow (+7.9 − (−7.9) %FM), and the equation resulted in a small mean positive bias of +0.0128 ± 3.9.

## Discussion

We report a field tool to predict body composition in women of South Asian ancestry, which uses triceps skinfold and weight measurements. The validity of this equation to predict %FM both for field use and for research purposes in women is indicated by the high correlation between the calculated %FM from the equation and %FM by the reference method (^2^H_2_O dilution). The strength of the new equation was the small bias and narrow limit of agreement between the actual and predicted values. Our values for the limit of agreement (+6.8 %FM) are better than those reported elsewhere [21].

The importance of this equation is in the finding that existing SFT prediction equations were not valid for estimating %FM in Sri Lankan women. Since there were no SFT equations for prediction of FM% developed in South Asian adult female populations, existing equations relevant to our population selected to test for validity were Siri [10], Brozek et al [11], and Durnin and Womersley equations [12]. The calculation of %FM by both Siri and Brozek et al. equations requires the calculation of body density prior to FM calculation. These published equations correlated well (*R* = 0.562–0.624, *p* < 0.001) with the reference method, yet, significantly underestimated %FM in Sri Lankan urban women, making them less suited for use in this population. The risk of using older equations developed on populations whose characteristics may be different to that in the new population has been demonstrated by Leahy et al. [22] who applied Durnin and Womersley equations to a current population and found that it was less accurate at higher body fat levels. Durnin and Womersley developed their equations in an era when body fat levels were lower on average in a given population. Such differences in body fat are also seen in different ethnic groups. Further, previous validation studies among Sri Lankan adolescents and children reported disagreement between actual and predicted body fat values, necessitating the development of new equations [13, 14].].

Differences in body proportion in Europeans and South Asians may account for underestimation observed in the present study when using existing equations. Durnin and Womersley equation [12] was developed in an age and gender stratified sample which included participants between 16 and 72 years who were moderately sedentary males and females with some participants being recruited from obesity clinics. The ethnic difference as well as the body composition differences in the populations could account for its inapplicability in our population. Due to ethnic similarity, a published equation for Sri Lankan adolescent girls was tested in this sample [13]. Here, the underestimation could be due to comparatively higher age, mean weight, BMI, and SFTs in adult women in this study.

The need to accurately assess body composition including body fat in South Asians is due to Asians having higher FM for a given BMI compared to Caucasians and the rising prevalence of non-communicable diseases including diabetes mellitus and overweight in Asia, and its link with body fat. Hence, the negative consequence of underestimation of FM during screening of a population is the resultant loss of access to preventive care. The significance of our new equation is in filling this gap of the requirements for accurate and precise measurement of FM in the general population. Our equation being developed in a current population has addressed the concerns of issues arising due to secular trends in body fat levels in populations.

While the simplicity of our equation is due to there being only a single skinfold measurement and weight, the strength of our equation is that triceps and weight measures were retained following the inclusion of four skinfold thickness measurements in generating the equation using regression. Further, as Freedmen highlights in his discussion of Bergman’s body fat index, studying body fat in men and women separately is important due to the difference in distribution of body fat [8, 23]. The new equations for women that were developed in Leahy et al.’s population included abdominal, biceps, triceps, mid-axillary, and medial calf [22]. These equations were developed using a wide range of SFT and girth measurements, and one could expect very high accuracy. Our limits of agreement for %FM compare well with these recent equations of Leahy and co-workers [21, 22]. Thus, for developing countries for use as a field tool, our equation has the simplicity of only having one SFT measurement and weight while preserving accuracy.

The narrow age range of participants may reduce generalizability of the results to women of reproductive age, without further validation and can be considered a limitation. However, the mean BMI of our sample is close to the mean BMI (22.8 ± 4.5 kg/m^2^) of a nationally representative study population [24]. Further, the BMI range of our population was 15.2–35.9 kg/m^2^ which reflects BMI observed in Sri Lanka, where morbid obesity is rare. Populations in other South Asian countries also show similar mean BMI values as reported here [25]. Hence, our sample reflects adequate variability, allowing applicability to such populations.

## Conclusions

Percentage FM by our SFT prediction equation significantly correlated with the reference method and provided excellent agreement with the reference measure. The feasibility for field use of this equation is enhanced by the fact that it uses only two parameters, namely triceps skinfold and weight measurements. The equation is suitable for use among urban Sri Lankan women and is possibly appropriate to be used in South Asian females, including women of South Asian origin living in Western countries.

## Abbreviations

^2^H_2_O: Deuterium dilution
BMI: Body mass index
FFM: Fat free mass
FM: Fat mass
FTIR: Fourier transform infrared
ISAK: International Society for Advancement of Kineanthropometry
SEE: Standard error of estimate
SFT: Skinfold thickness
SPSS: Statistical Package for Social Sciences
TBW: Total body water
WC: Waist circumference
WHO: World Health Organization
